# Polymorphisms in *Plasmodium falciparum* K13-Propeller in Angola and Mozambique after the Introduction of the ACTs

**DOI:** 10.1371/journal.pone.0119215

**Published:** 2015-03-19

**Authors:** Carlos Escobar, Sara Pateira, Elsa Lobo, Lis Lobo, Rosa Teodosio, Fernanda Dias, Natercia Fernandes, Ana Paula Arez, Luis Varandas, Fatima Nogueira

**Affiliations:** 1 Departamento de Pediatria, Hospital Prof. Doutor Fernando Fonseca EPE, Amadora, Portugal; 2 Unidade de Ensino e Investigação de Clínica Tropical (UEICT), Instituto de Higiene e Medicina Tropical, Universidade Nova de Lisboa, Lisbon, Portugal; 3 Unidade de Parasitologia Médica, Instituto de Higiene e Medicina Tropical, Universidade Nova de Lisboa, Lisbon, Portugal; 4 Departamento de Ciências Fisiológicas, Faculdade de Medicina, Universidade Eduardo Mondlane, Maputo, Moçambique; 5 Centro de Malária e outras Doenças Tropicais (CMDT), Instituto de Higiene e Medicina Tropical, Universidade Nova de Lisboa, Lisbon, Portugal; 6 Departamento de Medicina Interna, Faculdade de Medicina, Universidade Agostinho Neto, Luanda, Angola; 7 Departamento de Pediatria, Faculdade de Medicina, Universidade Eduardo Mondlane, Maputo, Moçambique; Institute of Tropical Medicine, JAPAN

## Abstract

We report the presence of SNPs in *Plasmodium falciparum* K13-propeller gene in two African countries, Angola and Mozambique, where malaria is a serious public health problem. Samples were collected before and after ACT introduction as first-line treatment. In each country 50 samples collected before and 50 after ACT introduction were analysed. A total of three different mutations (R471R and R575R in Angola and V494I in Mozambique) were identified in five samples, all collected after the introduction of ACT. The R471R mutation detected in Angola has already been reported in Africa (DR-Congo and Gabon). However, the mutations R575R (Angola) and V494I (Mozambique), have never been reported. V494I is adjacent to the known K13 resistance-associated mutation Y493H, although functional analysis did not predict a deleterious effect on protein function.

## Introduction

The emergence of artemisinin resistance is of great concern since the current first-line treatment for *Plasmodium falciparum* malaria in endemic countries relies on artemisinin-based combination therapies (ACT) [1]. Partial artemisinin resistance has been defined as a slower parasite clearance rate following treatment with an artesunate monotherapy, or after treatment with an (ACT) [1] and is increasingly common in South East Asia. Nevertheless this definition is affected by multiple factors such as patient immunity, blood drug concentration or partner drug activity [1]. Clinical response to ACT treatments (treatment failures) reflects the efficacy of both drugs in the combination, being the proportion of patients remaining parasitemic on day 3 the indicator of choice, currently used in routine monitoring for suspected artemisinin resistance [1]. Treatment failures observed in South East Asia correlate either with lower efficacy of the partner drug and with slower parasite clearance rate due to artemisinin partial resistance [2]. Historical evidence shows that the emergence of drug-resistant (chloroquine and sulphadoxine-pyrimethamine) *P. falciparum* strains first occurred in Southeast Asia, spread to East Africa and then spread outwards to the rest of the African continent [3].

During the last decade, African countries have gradually changed first-line treatment for uncomplicated *falciparum* malaria to ACT due to the development of resistance to the successively introduced anti-malarial drugs. In 2002, Mozambique (East Africa) replaced chloroquine by sulphadoxine-pyrimethamine in combination with amodiaquine (AQ-SP); in 2004 artesunate substituted AQ (AS-SP) and since 2009, this combination changed to artemether-lumefantrine [4]. In Angola (West Africa), the artesunate-amodiaquine combination was officially adopted in 2004, followed by artemether-lumefantrine in 2006 [5] and ACTs have been used nationwide since 2007.

In 2014, Ariey *et al*. [6] associated the artemisinin resistant phenotype to mutations on the kelch propeller protein K13 encoded in PF3D7_1343700 gene. In Cambodia, where these polymorphisms were first described, the presence of K13 mutants (mainly Y493H, R539T, I543T and C580Y) are correlated to *in vitro* parasite survival rates and *in vivo* parasite clearance rates. In August 2014, the World Health Organization included K13 mutations in the new working definition for partial artemisinin resistance stating that the presence of ≥5% of patients carrying K13 resistance-associated single nucleotide polymorphisms (SNPs) defines suspected artemisinin resistance; when K13 mutants are associated with either persistent parasitemia on day 3 or longer parasite clearance, resistance is confirmed [1].

K13-propeller SNPs have been searched in multiple locations aiming to describe the presence of resistant parasites before clinical resistance spreads, following the recommendations of WHO. To date, more than 60 mutations in K13 gene have been described in Africa and South East Asia [2,6,7–10], however, no resistance-associated mutations have been found outside the Greater Mekong Area [1].

In this study we report the presence of polymorphisms in *P. falciparum* K13-propeller in Angola and Mozambique, two portuguese-speaking countries endemic for malaria, before and after the introduction of ACT as first-line treatment.

## Methods

### Ethics statement

DNA samples were collected following individual oral consent and signed informed written consent from all participants or their guardians. Ethics approval to use the samples was obtained from: Comité de Ética do Ministério de Saúde de Angola (Committee of Ethics of the Ministry of Health of Angola), Comité de Ética do Hospital Pediátrico Dr. David Bernardino (Committee of Ethics of the Pediatric Hospital Dr. David Bernardino in Luanda Angola) and Comité Nacional de Bioética para a Saúde (CNBS) from the do Ministy of Health of Mozambique.

A set of 200 DNA samples from *P. falciparum*-infected individuals, already available from other studies [11–13], were analysed. Four groups of samples (50 each) from Angola and Mozambique, corresponding to time periods before and after the official introduction of ACT as the first-line treatment of malaria in both countries. In Angola, samples were collected during 2003 and during 2010. In Mozambique, one set of samples was collected between June 2003 and June 2005 and another set of samples between March 2010 and March 2012. All ethical aspects related with the sets of samples are described in the respective reports [11–13]. Samples were conserved as extracted DNA frozen at -20°c at *Instituto de Higiene e Medicina Tropical* in Lisbon (Portugal). A 520bp region (corresponding to codons 460 to 592) of PF3D7_1343700 gene was amplified by PCR. This region covered the region of the gene were the previously described SNPs associated with artemisinin resistance are located [6] and included in WHO definition [1] allowing the identification of the referred SNPs in one sequencing run.

Specific primers were developed for this purpose (forward—GTGTAGAATATTTAAATTCG; reverse—GCTCCTGAACTTCTAGCTTC). An aliquot of the PCR products were analyzed by electrophoresis on a 2% agarose gel stained with ethidium bromide to confirm amplification. A second semi-nested procedure was performed in some samples, obtaining a fragment of 445bp (reverse primer—GCTCCTGAACTTCTAGCTTC). Products were sequenced at STAB VIDA Genomics LAB, Caparica, Portugal) and aligned to 3D7 reference strain using Multalin software (http://multalin.toulouse.inra.fr/; free online). Samples were SNPs were detected underwent new PCR amplification and sequencing procedure to ascertain the results. The genotype data used in the analysis have been submitted to the National Center for Biotechnology Information for public access (accession numbers: KP262063, KP262064, KP262065, KP262066 and KP262067).

In order to predict biological functional relevance for non-synonymous SNPs we used PROVEAN software tool (http://provean.jcvi.org/; free online). This software is validated to predict whether a protein sequence variation (substitution, deletion or insertion of single or multiple amino acid) affects protein function in human and non-human proteins on an homology-based approach. The algorithm uses a sequence homolog collected from the NCBI database through BLAST. In our study human KEAP1 protein was used as reference (PDB ID: 3VNG) as previously described by Ariet et al. [6]. PROVEAN algorithm assumes a score of-2.5 as cut-off for a binary neutral/deleterious function classification with a sensitivity of 80.4% and a specificity of 78.6%.

## Results

### P. falciparum K13-propeller polymorphisms observed

**Fig 1:**
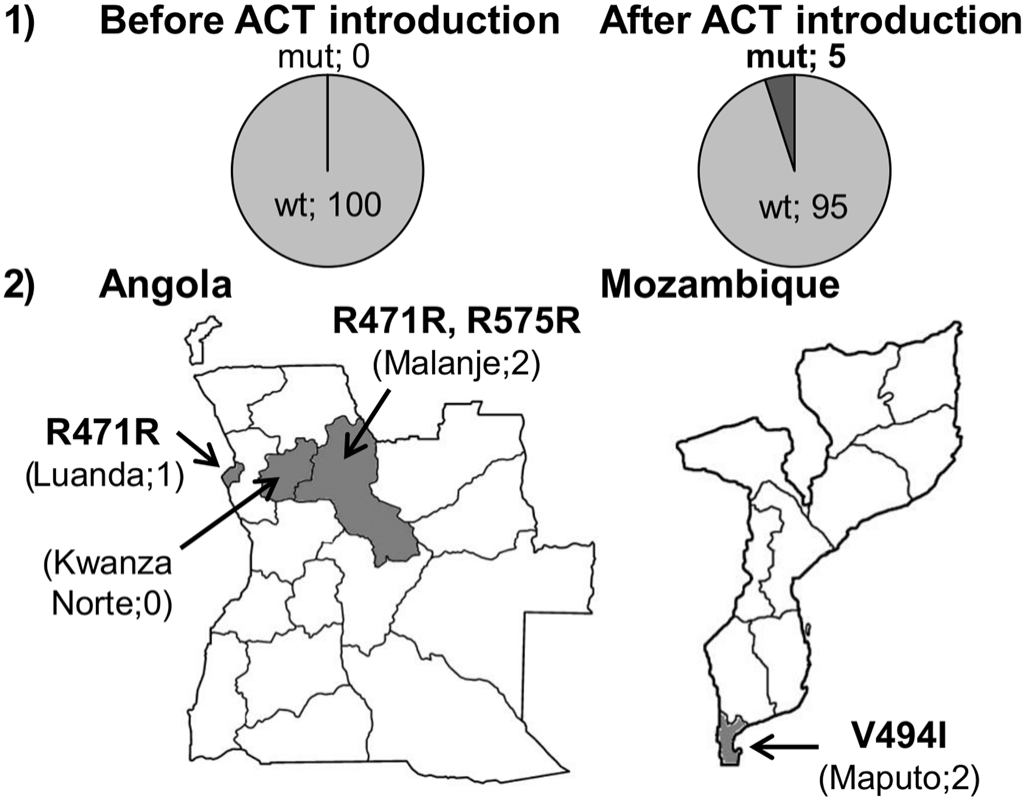
*P. falciparum* K13-propeller polymorphisms prevalence and distribution in Angola and Mozambique. Panel 1) Proportion within each time period of wild type (wt) and mutant (mut) samples; Panel 2) Geographical location of sample collection (dark grey) and identified polymorphisms.

A total of 3 different mutations (R471R, V494I, R575R) were found in 5 samples (2.5%). All mutations were found in samples collected after the introduction of ACTs as the official first-line treatment in each country. Fig. 1 shows geographical origin of the samples and time period, and Table 1 shows K13-propeller polymorphisms identified.

**Table 1:** *P. falciparum* K13-propeller amino acid and nucleotide substitutions observed in Angola and Mozambique.

Locusa.a.	Locus Nuc.	Ref. codon	Mut. codon	Country—Region (n)
R471R*	1413	CGT	CGC	Angola—Malanje (1) & Luanda (1)
V494I	1483	GTT	ATT	Mozambique—Maputo (2)
R575R	1725	AGA	AGG	Angola—Malanje (1)

*SNP has been previously observed in DR Congo, Kinshasa [9] and Gabon, Libreville [10]. All data are relative to reference sequence PF3D7_1343700. Abbreviations: a.a., aminoacid; nuc, nucleotidic; ref, reference; mut, mutated; n, number of samples containing mutant allele.

The newly described polymorphism V494I was found in two samples from Maputo province (Mozambique). Two synonymous polymorphisms (R471R in two samples and R575R in one sample) were found in Angola. R575R, also newly described, was found in Malanje, an Angolan province that borders the Democratic Republic of Congo. R471R (found in Luanda and Malanje) has already been observed in DR Congo (Kinshasa) [9] and Gabon (Libreville) [10]. All polymorphisms were uncommon (R471R 4%, V494I 4%, R575R 2%), and each was present in different isolates. The mutations associated to artemisinin-resistance (Y493H, R539T, I543T and C580Y) described in Southeast Asia [2,6] were not detected.

### Functional assessment of polymorphisms observed

Using PROVEAN software tool with a cut-off score of-2.5, we predicted that V494I variant may have a neutral effect on protein biological function (PROVEAN score = -0.774). The same cut-off for known polymorphisms in K13-propeller predicted a deleterious effect on protein function for all (Y493H score = -4.166, I543T score = -3.401, and C580Y score = -2.849) except for R539T which falls right below significance (score = -2,489).

## Discussion

The shift in drug policy in Africa towards ACTs was recent, it has been accompanied by both the selection of polymorphisms associated with decreased lumefantrine sensitivity [13,14] and by the decrease of the *in vitro* sensitivity to this drug [15]. The reduced sensitivity to ACT partner drugs may increase the selection of artemisinins resistant parasites. Thus, the use of the new molecular marker K13-propeller is fundamental for surveillance in malaria control programs, in order to prolong the life span of the ACTs in Africa.

Our study on *P. falciparum* K13-propeller polymorphisms showed the presence of three mutations, two of them not previously described. One of the mutations is a non-synonymous SNP (V494I) that lays one codon next to the known K13 resistance-associated mutation (Y493H). Although functional analysis for V494I did not predict deleterious effect on protein function (PROVEAN score = -0,774) as it does for Y493H (score = -4,166), this does not necessarily mean that the protein is not affected. For instance, R539T was considered neutral in a similar analysis (score = -2,489), although it is associated to ACT drug response in Southeast Asia [6]. Plus, V494I was identified in two samples collected in Mozambique after the introduction of ACTs. Mozambique is located at East Africa, a region recognized as being a major focus of drug resistant parasites entrance and development in sub-Saharan Africa, probably originated at Southeast Asia [3]. While speculative, justifies further attention. The other two polymorphisms described in our study encode synonymous substitutions, thus not affecting protein structure and function. Mutation R471R observed in samples collected after ACT introduction in Malanje and Luanda provinces (Angola) has also been reported in two other western African countries, DR Congo [9] and Gabon [10], however it is difficult to attribute this to the spread of a *P. falciparum* clone or to multiple emergence of this SNP. Nevertheless, parasites in endemic regions of sub-Saharan Africa are less likely to be clonal because of the high transmission intensity [10]. Recently, K13 resistance-associated mutations have been demonstrated to arise both population-specific and independently in different geographic areas in Southeast Asia [2]. Multiple non-synonymous polymorphisms have been published, but there are very few shared mutant alleles between African and Southeast Asia parasites as yet [2,6,9].

The absence of K13 resistance-associated mutants from Southeast Asia in Africa [7–10] is promising, but continuous tracking of resistance emergence should be implemented enabling early detection of resistant parasites. The use of a molecular marker such as K13 mutations is nowadays a cornerstone for malaria surveillance programs, but differences between African and Asian K13-mutant parasites should be taken into account, and this study provides useful information on two not previously described K13 polymorphisms in Angola and Mozambique where malaria is a serious public health problem.

Whether or not the mutations identified during the present study are associated to drug response, can not be inferred from our results (no phenotype of drug resistance was observed in the mutant carrier samples). Nevertheless, no SNPs were present in the samples collected before the introduction of the ACTs as a first-line of treatment in each country.
